# Prognostic factors affecting survival after surgical resection of gastrointestinal stromal tumours: a two-unit experience over 10 years

**DOI:** 10.1186/1477-7819-4-73

**Published:** 2006-10-09

**Authors:** Antonio Chiappa, Andrew P Zbar, Michael Innis, Stuart Garriques, Emilio Bertani, Roberto Biffi, Giancarlo Pruneri, Felipe Luzzato, Paolo Della Vigna, Cristina Trovato, Bruno Andreoni

**Affiliations:** 1 Dept of General Surgery, European Institute of Oncology, University of Milan, Italy; 2 Dept of General Surgery, School of Clinical Medicine and Research, University of the West Indies, Barbados; 3 Dept of Pathology, The University of the West Indies, Queen Elizabeth Hospital, Barbados; 4 Dept of Pathology, European Institute of Oncology, University of Milan, Italy; 5 Dept of Radiology, European Institute of Oncology, Milan, Italy; 6 Division of Endoscopy, European Institute of Oncology, Milan, Italy

## Abstract

**Background:**

Gastrointestinal stromal tumours (GISTs) are the most common mesenchymal neoplasm of the gastrointestinal (GI) tract which has only been recently described based on their specific immunohistochemistry and the presence of particular KIT-related mutations which potentially make them targets for tyrosine kinase inhibition.

**Methods:**

Sixty-one patients (29 M; 32 F, median age 60 years; range: 23–86 years) between June 1994 and March 2005, were analyzed from two allied institutions. Patient, tumour, and treatment variables were analyzed to identify factors affecting survival.

**Results:**

Of the 61 patients, 55 (90%) underwent complete surgical resection of macroscopic disease. The 5-year overall survival (OS) rate in the 61 patients was 88% and the 5-year disease-free survival (DFS) in the 55 cases completely resected was 75%. Univariate analysis revealed that R0 resection was strongly associated with a better OSrate (p < 0.0001). Likewise, univariate analysis also showed high mitotic count of > 10 mitoses/per 50 HPF was a significant variable in worse prognosis for OS (≤ 10 mitoses/per 50 HPF 95% 5-year OS vs. > 10 mitoses/per 50 HPF 74% 5-year OS, respectively; p = 0.013). On subsequent multivariate analysis, only high mitotic count remained as a significant negative prognostic variable for OS (p = 0.029). Among patients resected for cure, there were 8 recurrences during follow-up. The mean time to recurrence was 21 ± 10 months (range: 4–36 months). Univariate analysis revealed that mitotic count of > 10 mitoses per 50 high power fields, intratumoural necrosis, and pathological tumour size (> 10 cm in maximal diameter) significantly correlated with DFS (p = 0.006, 0.002 and 0.02, respectively), with tumour necrosis and high mitotic count remaining as independent predictive variables affecting prognosis on subsequent multivariate analysis.

**Conclusion:**

Most GISTs are resectable with survival principally dependent upon mitotic count and completeness of resection. Future metabolic and genetic analyses will define the role of and resistance to induction or postoperative adjuvant targeted kinase inhibition therapy.

## Background

Gastrointestinal stromal tumors (GISTs) are uncommon tumours which have relatively recently been separated from conventional leiomyomas, leiomyoblastomas, and leiomyosarcomas, based on their specific immunohistochemistry, presumed aetiopathogenesis, molecular biology and differential outcome [1,2]. There is still controversy over their histogenetic origin with immunohistochemical and ultrastructural resemblance to the interstitial cells of Cajal [3,4] as well as from cells which are smooth muscle cell precursors responsible for generation of the slow-wave pacemaker activity of the gut musculature [5]. The designation of these tumours has largely been based on the immunohistochemical expression of c-KIT (CD 117; stem cell factor [6,7], and CD 34) [8], with a relative lack of desmin and S-100 immunoreactivity, although recently, a subset of KIT-negative GISTs has been recognised with the morphological features of classical stromal tumours but retaining PKC-theta expression [9]. More recently, cytogenetic and comparative genomic hybridisation (CGH) studies have shown characteristic chromosomal patterns in GISTs which have distinguished them from other gastrointestinal mesenchymal tumours, [10] and where most GISTs demonstrate specific DNA copy number changes which correlate with their clinical behaviour and which differentiate benign from malignant cases [11].

Considerable debate still exists concerning the biological behaviour of GISTs, centering largely around the tumour site, tumour size, the presence of intra-tumoural necrosis, and mitotic count [12-14]. Based on this recent data, our study assesses the surgical outcome of unselected GISTs referred to two allied institutions over a 10 year period and analyses the factors affecting prognosis after resection with curative intent.

## Patients and methods

### Patients

Using our hospital databases, we collected the records of patients with a histopathologic diagnosis of primary leiomyoma, leiomyoblastoma, leiomyosarcoma, gastrointestinal sarcoma, and stromal cell tumour of the GI tract from the period including 1994 to October 2004. Each of the patients had undergone surgical resection of their tumour. We recorded the patients' age, gender, presenting clinical symptoms, tumour site, maximal tumour diameter after resection, duration of surgery, surgical procedure, extent of surgical resection, the presence and date of local recurrence or distant metastasis, and the clinical outcome until last follow-up, including date of death where appropriate. There was no strict follow-up protocol in either institution, although follow-up included physical examination, chest X-ray, thoracoabdominal and pelvic computerised tomography (CT) scan, endoluminal ultrasonography, and GI endoscopy, where appropriate. The median follow-up of all patients was 35 months (range 2–140 months). Those tumours deemed to have histologically free resection margins were included as cases where surgery was performed with curative intent and were referred to as complete (R0) resections. After the surgical treatment, patients were followed regularly using clinical assessment on a 3 monthly basis with abdominopelvic CT and/or ultrasound annually, where appropriate.

### Immunohistochemistry

The tumour samples from all 61 patients were evaluated for various markers by using immunohistochemistry with commercially available antibodies against CD 117-KIT, (1:50, Santa Cruz Biotech, CA USA) S-100 protein, (1:40, Novocastra Labs, USA) Desmin, (1:50, DAKO, Glostrup, Denmark) and Smooth-muscle Actin (1:200, DAKO, Glostrup, Denmark) with qualitative assessment of immunoreactivity in accordance with the manufacturers' instructions. Cases were classified according to risk and their potential for aggressive clinical behaviour based on the NIH consensus statement of 2001 for GISTs, [15] where tumour size < 2 cm and mitotic count < 5/50 high power fields (HPF) was graded as very low risk, tumour size between 2 and 5 cm and a mitotic count < 5/50 HPF was considered low risk, tumours < 5 cm with a mitotic count between 5/50 HPF and 10/50 HPF or tumours between 5 and 10 cm with a mitotic count less than 5/50 HPF were deemed as intermediate risk and tumours > 10 cm and/or those tumours with a mitotic count > 10/50 HPF and tumours > 5 cm with a mitotic count > 5/50 HPF were classified as high risk.

### Statistics

Statistical analysis was performed with SPSS 12.0 software (Chicago, IL, USA). The Student's t test was used to compare continuous variables with the χ^2 ^test being utilized for dichotomous variables. Overall survival was calculated from the day of diagnosis until death or the last day of a patient's visit to the outpatient clinic. The disease-free survival was calculated from the first diagnosis until tumour recurrence or distant metastases were found. Kaplan-Meier analysis with a Mantel-Haenszel log-rank test was used to compare overall and recurrence-free survival [16] with the proportional hazards method being used to evaluate significant prognostic factors [17]. All p values < 0.05 are reported.

## Results

Of the resected cases, there were 29 males and 32 females with an overall median age 60 years (range 23–86 years). All tissue samples were proven to be CD-117 positive on immunohistochemical staining. Tumours were located in the stomach in 41 cases (67%), the small bowel in 14 (23%), the colon in 4 (5%) the rectum in 1 (2%), and the duodenum in 2 cases (3%). Three of the 61 patients (5%) presented with synchronous hepatic metastases. The most frequent presenting symptoms included gastrointestinal tract bleeding and abdominal pain in 11 cases (18%) and abdominal fullness and/or discomfort in 7 patients (11%). Twenty patients (33%) had anaemia on presentation with 15 patients (26%) having a palpable abdominal mass on clinical examination. Six patients (10%) were asymptomatic and were referred for incidental GISTs after routine physical examination and investigation.

The surgeries performed for the patient cohort are shown in Table 1. One patient with a gastric GISTs and a single synchronous hepatic metastasis in segment III underwent a non-anatomical wedge resection of the liver at the same time as their gastric resection. Postoperative complications included gastrointestinal hemorrhage in one case, duodenal leakage in a further patient, and urosepsis in one case. Perioperative 30 day mortality was 0 per cent. Pathological assessment of the resected tumours showed a median tumour size of 5.3 cm (range, 0.6–38 cm). Ten tumours (16%) were < 2 cm in maximal diameter, 19 (31%) between 2–5 cm, 17 (28%) between 5–10 cm and 15 (25%) > 10 cm. The mitotic counts were < 5/50 per HPF in 37 cases (61%), between 5 and 10/50 per HPF in a further 6 (10%) and > 10/50 per HPF in 18 (29%) cases. Ulceration of the tumour was noted in 69% (42/61), haemorrhage in 25% (15/61), and tumour necrosis in 28% (17/61). Six patients (10%) underwent palliative surgery because of tumour bleeding resulting in severe anaemia, 3 patients presented with synchronous resectable hepatic metastases, and a further 3 presented with an unresectable intra-abdominal tumour. According to the NIH Consensus Conference criteria, of all cases there were 10 patients (16%) in the very low risk class, 15 cases (25%) in the low risk class, 7 (11%) in the intermediate risk class, and 29 (48%) in the high risk class. Table 2 shows the clinico-pathological data of patients according to the number of mitoses per HPF (i.e. ≤ or > 10/50).

**Table 1:** Surgical procedures performed in 61 patients affected by gastrointestinal stromal tumours.

**Surgical procedure**	**N (%)**
Wedge gastric resection*	31 (51)
Small bowel resection	14 (23)
Subtotal gastrectomy	8 (13)
Colonic resection	3 (5)
Total gastrectomy	2 (3)
Duodenum-pancreatectomy	2 (3)
Transanal excision	1 (2)

*One patient underwent non-anatomical wedge liver resection at the same time as the gastric resection for an isolated liver metastasis in segment III.

**Table 2:** Clinico-pathological variables of 61 gastrointestinal stromal tumours patients according to number of mitoses per 50 HPF(≤ or > 10)*

	Mitoses ≤ 10/50 per HPF	**Mitoses > 10/50 per HPF**	**p**
**Tumour location**			0.09
stomach	28 (68)§	13 (32)	
colon-rectum	1 (25)	3 (75)	
small bowel	12 (86)	2 (14)	
duodenum	2 (100)	0	
			
**Surgical resection type**			**0.04**
R0	42 (74)	15 (26)	
R1+R2	1 (25)	3 (75)	
			
**Tumour diameter**			0.71
≤ 10 cm	33 (72)	13 (28)	
> 10 cm	10 (67)	5 (33)	
			
**Tumour necrosis**			
absent	33 (75)	11 (25)	
present	10 (59)	7 (41)	0.21

*HPF, high power field

§Numbers in parentheses are percentages.

**Figure 1:**
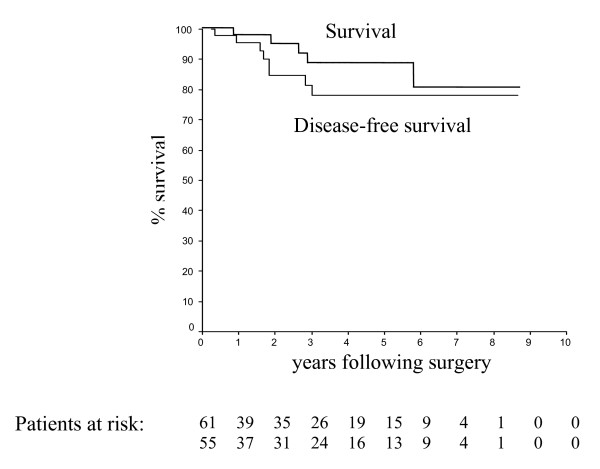
Overall survival and disease-free survival in 61 patients with GIST tumours.

**Figure 2:**
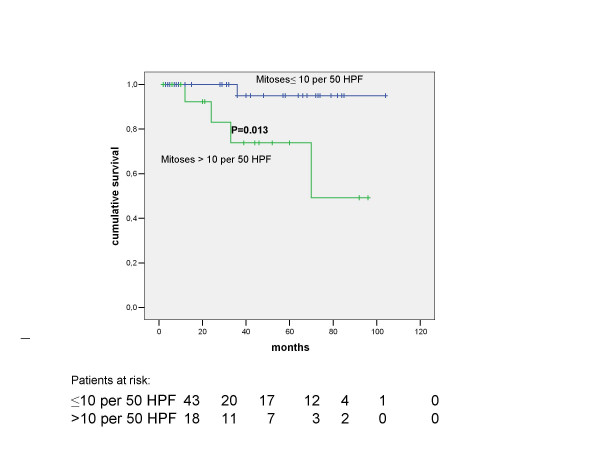
Overall survival according to number of mitoses for the 61 patients with GIST tumours.

Of the 61 patients resected, 55 (90%) had their tumour completely resected as an R0 resection as described in the methods section. The 5-year overall survival rate for these patients was 88%, compared with 0% (0/6) for those patients who underwent palliative surgery (p < 0.0001). During the follow-up period, 5 patients died of recurrent disease, all of whom were in NIH class 4 on initial presentation. A further 2 patients died of myocardial infarction during the follow-up period but were free of tumour at the time of their deaths. Seven patients who experienced recurrence were treated with Imatinib 400 mg daily and 6 of them are still alive after a median follow-up period of 18 months. Figure 1 shows the overall and disease-free survival of all GISTs in the series undergoing all types of resections. On univariate analysis, histological resection-free margins (i.e., R0 resection margins) were strongly associated with improved overall survival (91% 5-year overall survival for complete resection vs. 0% for incomplete resections, p < 0.0001). This effect was preserved in patients presenting with advanced classes of disease, where amongst the NIH class 4 patients completeness of resection correlated with overall survival (83% 5-year overall survival for complete resection vs. 0% 5-year overall survival for incomplete resection, p = 0.0031). The other significant factor affecting overall survival on univariate analysis was the number of mitoses (> 10 per 50 HPF) where there was a 95% 5-year overall survival for patients with a mitotic count ≤ 10 per 50 HPF vs. 74% 5-year overall survival for patients with a mitotic count > 10 per 50 HPF, p = 0.013) (Figure 2). Multivariate analysis of these two significant factors detected on univariate analysis revealed that the number of mitoses (> 10 per 50 HPF) was the only significant independent predictive variable affecting overall survival (HR = 12.350; CI 1.295–117.781: p = 0.029).

The 5-year disease-free survival in the 55 patients completely resected was 75% (Figure 2). Among those patients resected for cure, 8 patients developed tumour recurrence after a mean of 21 ± 10 months (range: 4–36 months) follow-up. The liver was the most frequent site of tumour relapse (n = 6; 75% of the recurrences) and in the 8 patients, survival after recurrence was 30% at 3 years. Patients designated as NIH class 4 had a 5-year disease-free survival rate of 65% compared with 94% for all other groups combined (p = 0.03). On univariate analysis, a higher number of mitoses (i.e. > 10 per 50 HPF) correlated with worse disease free-survival in those resected for cure (87% for < 10 mitoses/per 50 HPF *vs*. 57% for > 10 mitoses/per 50 HPF respectively, p = 0.006), as did tumour size (tumours < 10 cm in maximal diameter had an 86% disease-free survival at 5 years compared with only 63% for those tumours > 10 cm in size; p = 0.02). The presence of tumour necrosis was also significantly associated with a worse disease-free survival on univariate analysis (51% vs. 91% 5-year disease-free survival with or without tumour necrosis respectively; p = 0.002). On multivariate analysis of these three significant factors detected on univariate analysis, only tumour necrosis and the number of mitoses (> 10 mitoses/per 50 HPF) proved to be significant independent predictive variable affecting disease-free survival (HR = 11.226; CI 2.199–57.315: p = 0.004 and HR = 8.054; CI 1.842–35.205: p = 0.006, respectively).

## Discussion

Analysis of 61 cases of mixed-site GISTs in our study obtained from two tertiary referral centres over a 10-year period reveals that both overall survival and disease-free survival are affected by a high mitotic count in the tumour after resection with curative intent. The primary tumour sites noted in our study are similar to other previous reports [18,19], with a similar demographic of presenting symptoms including gastrointestinal bleeding, abdominal pain and the presence of an abdominal mass [20-22]. CT and MRI are generally used for the preoperative imaging of GISTs [23-25]. However, there are no well-established, published guidelines to help surgeons determine the optimal manner in which to evaluate this intraabdominal, site-specific class of tumours. Although not reported in the results section of our current report, our limited use of endoluminal ultrasound has been unable to assist in the definition of malignant versus benign cases. Nevertheless, this has been as previously described [26], where tumour margin irregularity and heterogeneity with intratumoural degeneration and larger tumour size were more indicative of malignancy. No patient in our series underwent preoperative image-guided biopsy or aspiration cytology as has been advocated by some authors [27]. Lastly, recent data shows a potential value for preoperative positron emission tomography (PET) in the prediction of malignant potential with a correlation between ^18^FDG uptake and mitotic count [28]

Others have shown that one of the principal prognostic factors determining cancer-specific outcome is tumour size, where tumours > 10 cm in maximal diameter have a relatively worse survival following surgical resection [2,12,18]. Our study was unable to substantiate an effect of tumour size except on univariate analysis of overall survival. This effect was not mantained on multivariate analysis, although larger patient numbers are probably required to determine a true effect of tumour size on cancer-specific outcomes. In this respect, GISTs, which exceed 5 cm in maximal diameter, have been previously shown to correlate with **a **worse prognosis independent of their primary location, [19,29] where tumour diameter is part of most prognostic indices designed to govern treatment algorithms for patients deemed at high risk [2,15,30]. Equally, as in our study, most reports have shown high mitotic count to equate with a worse tumour-specific outcome [12,31], although some recent studies have excluded this factor along with that of intratumoural necrosis from model analyses since they have not been proven to be independent prognostic markers of survival [12,32]. Our study showed an effect of intratumoural necrosis on disease-free but not overall survival. Other studies have confirmed more objective immunohistochemical evaluation of cellular proliferation as correlating with an overall worse outcome using Ki 67 analysis, [2] proliferating cell nuclear antigen labelling index, cellular apoptotic marker (Bcl-2 and Bax) expression and overall tumour cellularity estimates [12,33,34].

**T**he clinicopathologic evaluation of GISTs is particularly difficult, since these tumours have only recently been characterized and since some previous studies have still grouped these tumours with spindle-cell and epithelial neoplasms, which may share similar histopathology, but which possess divergent outcomes. Attempts have been made to categorize these tumours by the establishment of an individualized immunophenotype based on neural and smooth muscle immunodifferentiation although up to one-quarter of these neoplasms cannot adequately be categorized on this basis [35]. No patient in our series was treated primarily with the tyrosine kinase inhibitor Imatinib, which has been shown to be valuable in metastatic kit-positive GISTs [36,37]. We have used this in our units only after proven disease recurrence. This issue is complex, since although most GISTs acquire a gain-of-function c-KIT mutation, many tumours ultimately demonstrate some resistance to targeted molecular therapy during treatment [38]. It appears that c-KIT gene mutations are pivotal molecular events in the life of these tumours, adversely affecting their overall prognosis after surgery [39]. Equally, there is controversy regarding the mechanisms of Imatinib resistance which are dependent upon missense mutations of the kinase domains of KIT and PDGFRA and there is no clear evidence at the present time that routine molecular analysis of GISTs to assess exon mutations alters management [40]. Moreover, the follow-up protocol for resected GISTs has not been established although there are relatively specific changes detectable in tumours treated primarily with Imatinib on CT and MR imaging, including changes in tumour size, attenuation, enhancement, and vascularity [41,42]

## Conclusion

Our and other studies suggest that complete surgical resection (R0 resection) of GISTs as well as their initial size, mitotic activity, and the presence of intratumoural necrosis are potentially important variables associated with overall outcome, with the principal factor affecting overall survival being the mitotic count. These findings have been suggested in another very recently published report [43]. In the era of Imatinib effectiveness, it is unclear what is the role of preoperative induction or postoperative adjuvant targeted therapy, as well as the place of surgery in partial Imatinib responsiveness or in isolated metastatic disease [44]. In this light, at the present time, it may be that a clinico-pathological interpretation of the risk status of the tumour, as used in our study, is the most practical approach for such decision where complete surgical resection (R0 resection) is the key prognostic factor [45] and where high-risk GISTs surgery is supported by a selective approach towards induction or postoperative adjuvant molecular therapy [46,47].

## Competing interests

The author(s) declare that they have no competing interests.

## Authors' contributions

**AC, **study conception and design and drafting of manuscript; **APZ**, critical revision, drafting of manuscript; **MI **acquisition of data; **SG**, performed immunohistochemistry; **EB**, analysis and interpretation of data; **RB**, acquisition of data; **GP**, performed immunohistochemistry; **FL **performed immunohistochemistry; **PDV**, acquisition of data; **CT, **acquisition of data; **BA**, critical revision.

## Funding source

None

## References

[B1] Suster S (1996). Gastrointestinal stromal tumours. Semin Diagn Pathol.

[B2] Langer C, Gunawan B, Schüler P, Huber W, Füzesi L, Becker H (2003). Prognostic factors influencing surgical management and outcome of gastrointestinal stromal tumours. Br J Surg.

[B3] Meittinen M, Sarlomo-Rikala M, Lasota J (1999). Gastrointestinal stromal tumours: recent advances in understanding their biology. Hum Pathol.

[B4] Sircar K, Hewlett BR, Huizinga JD, Chorneyko K, Berezin I, Riddell RH (1999). Interstitial cells as precursors of gastrointestinal stromal tumours. Am J Surg Pathol.

[B5] Kindblom LG, Remotti HE, Aldenborg F, Meis-Kindblom JM (1998). Gastrointestinal pacemaker cell tumour (GIPACT): gastrointestinal stromal tumours show phenotypic characteristics of the interstitial cells of Cajal. Am J Pathol.

[B6] Miettinen M, Sobin LH, Sarlomo-Rikala M (2000). Immunohistochemical spectrum of GISTs at different sites and their differential diagnosis with reference to CD117 (KIT). Mod Pathol.

[B7] Miettinen M, Lasota J (2001). Gastrointestinal stromal tumors – definition, clinical, histological, immunohistochemical and molecular genetic features and differential diagnosis. Virchows Arch.

[B8] Miettinen M, Virolainen M, Maarit-Sarlomo-Rikala (1995). Gastrointestinal stromal tumours–value of CD34 antigen in their identification and separation from true leiomyomas and schwannomas. Am J Surg Pathol.

[B9] Debiec-Rychter M, Wasag B, Stul M, De Wever I, Van Oosterom A, Hagemeijer A, Sciot R (2004). Gastrointestinal stromal tumours (GISTs) negative for KIT (CD117 antigen) immunoreactivity. J Pathol.

[B10] Gunawan B, Bergmann F, Hoer J, Langer C, Schumpelick V, Becker H, Fuzesi L (2002). Biological and clinical significance of cytogenetic abnormalities in low-risk and high-risk gastrointestinal stromal tumours. Hum Pathol.

[B11] El-Rifai W, Sarlomo-Rikala M, Andersson LC, Knuutila S, Miettinen M (2000). DNA sequence copy number changes in gastrointestinal stromal tumours: tumour progression and prognostic significance. Cancer Res.

[B12] Kontogianni K, Demonakou M, Kavantzas N, Lazaris ACh, Lariou K, Vourlakou C, Davaris P (2003). Prognostic predictors of gastrointestinal stromal tumours: a multi-institutional analysis of 102 patients with definition of a prognostic index. Eur J Surg Oncol.

[B13] Aparicio T, Boige V, Sabourin JC, Crenn P, Ducreux M, Le Cesne A, Bonvalot S (2004). Prognostic factors after surgery of primary respectable gastrointestinal stromal tumours. Eur J Surg Oncol.

[B14] Ozguc H, Yilmazlar T, Yerci O, Soylu R, Tumay V, Filiz G, Zorluoglu A (2005). Analysis of prognoistic and immunohistochemical factors in gastrointestinal tumours with malignant potential. J Gastrointest Surg.

[B15] Fletcher CD, Berman JJ, Corless C, Gorstein F, Lasota J, Longley BJ, Miettinen M, O'Leary TJ, Remotti H, Rubin BP, Shmookler B, Sobin LH, Weiss SW (2002). Diagnosis of gastrointestinal stromal tumours: a consensus approach. Hum Pathol.

[B16] Kaplan EL, Meier P (1958). Nonparametric estimation from incomplete observation. J Am Stat Assoc.

[B17] Peto R, Pike MC (1973). Conservation of the approximation (O-E2)/E in the log-rank test for survival data on tumour incidence data. Biometrics.

[B18] DeMatteo RP, Lewis JJ, Leung D, Mudan SS, Woodruff JM, Brennan MF (2000). Two hundred gastrointestinal stromal tumours: recurrence patterns and prognostic factors for survival. Am Surg.

[B19] Emory TS (1999). Prognosis of gastrointestinal smooth-muscle (stromal) tumours: dependence on anatomic site. Am J Surg Pathol.

[B20] Nishida T, Nakamura J, Taniguchi M, Hirota S, Ito T, Kitamura Y, Matsuda H (2000). Clinicopathological features of gastric stromal tumours. J Exp Clin Cancer Res.

[B21] Pardo Martinez C, Mayol Martinez J, Hernandez Perez C, Alvarez Fernandez-Represa J (2004). Gastric stromal tumours: clinical presentation and surgical options. Rev Esp Enferm Dig.

[B22] Nowain A, Bhakta H, Pais S, Kanel G, Verma S (2005). Gastrointestinal stromal tumours: clinical profile, pathogenesis, treatment strategies and prognosis. J Gastroenterol Hepatol.

[B23] Caramella T, Schmidt S, Chevallier P, Saint Paul M, Bernard JL, Bidoli R, Bruneton JN (2005). MR features of gastrointestinal stromal tumours. Clin Imaging.

[B24] King DM (2005). The radiology of gastrointestinal stromal tumours (GISTs). Cancer Imaging.

[B25] Tateishi U, Miyake M, Maeda T, Arai Y, Seki K, Haegawa T (2006). CT and MRI findings in KIT-weak and KIT-negative atypical gastrointestinal stromal tumours. Eur Radiol.

[B26] Palazzo L, Landi B, Cellier C, Cuillerier E, Roseau G, Barbier JP (2000). Endosonographic features predictive of benign and malignant gastrointestinal stromal cell tumours. Gut.

[B27] Li SQ, O'Leary TJ, Buchner SB, Przygodzki RM, Sobin LH, Erozan YS, Rosenthal DL (2001). Fine needle aspiration of gastrointestinal stromal tumours. Acta Cytol.

[B28] Kamiyama Y, Aihara R, Nakabayashi T, Mochiki E, Asao T, Kuwano H, Oriuchi N, Endo K (2005). 18 F-Fluorodeoxyglucose positron emission tomography: useful technique for predicting malignant potential of gastrointestinal stromal tumours. World J Surg.

[B29] Rudolph P, Chiaravalli AM, Pauser U, Oschlies I, Hillemanns M, Gobbo M, Marichal M, Eusebi V, Hofler H, Capella C, Kloppel G (2002). Gastrointestinal mesenchymal tumours – immunophenotypic classification and survival analysis. Virchows Arch.

[B30] Fraquemont D (1995). Differentiation and risk assessment of gastrointestinal stromal tumours. Am J Clin Pathol.

[B31] Bearzi I, Mandolesi A, Arduini F, Costagliola A, Ranaldi R (2006). Gastrointestinal stromal tumour. A study of 158 cases: clinicopathological features and prognostic factors. Anal Quant Cytol Histol.

[B32] Iaesalnieks I, Rummele P, Dietmaier W, Jantsch T, Zulke C, Schlitt HJ, Hofstadter F, Anthuber M (2005). Factors associated with disease progression in patients with gastrointestinal stromal tumours in the pre-imatinib era. Am J Clin Pathol.

[B33] Tarn C, Godwin AK (2005). Molecular research directions in the management of gastrointestinal stromal tumours. Curr Treat Options Oncol.

[B34] Nakamura M, Yamamoto H, Yao T, Oda Y, Nishiyama K, Imamura M, Yamada T, Nawata H, Tsuneyoshi M (2005). Prognostic significance of expressions of cell-cycle regulatory proteins in gastriointestinal stromal tumour and the relevance of risk grade. Hum Pathol.

[B35] Rubin B, Fletcher J, Fletcher C (2000). Molecular insights into histogenesis and pathogenesis of gastrointestinal stromal tumours. Surg Pathol.

[B36] Harrison ML, Goldstein D (2006). Management of metastatic gastrointestinal stromal tumour in the Glivec era: a practical case-based approach. Intern Med J.

[B37] Raut CP, Posner M, Desai J, Morgan JA, George S, Zahrieh D, Fletcher CD, Demetri GD, Bertagnolli MM (2006). Surgical management of advanced gastrointestinal stromal tumours after treatment with targeted systemic therapy using kinase inhibitors. J Clin Oncol.

[B38] Tarn C, Skorobogatko YV, Taguchi T, Eisenberg B, von Mehren M, Godwin AK (2006). Therapeutic effect of imatinib in gastrointestinal stromal tumours: AKT signaling dependent and independent mechanisms. Cancer Res.

[B39] Liu XH, Bai CG, Xie Q, Feng F, Xu ZY, Ma DL (2005). Prognostic value of KIT mutation in gastrointestinal tumours. World J Gastroenterol.

[B40] Koay MH, Goh YW, Iacopetta B, Grieu F, Segal A, Sterrett GF, Platten M, Spagnolo DV (2005). Gastrointestinal stromal tumours (GISTss): a clinicopathological and molecular study of 66 cases. Pathology.

[B41] Hong X, Choi H, Loyer EM, Benjamin RS, Trent JC, Charnsangavei C (2006). Gastrointestinal stromal tumour: role of CT in diagnosis and in response evaluation and surveillance after treatment with Imatinib. Radiographics.

[B42] Stroszczynski C, Jost D, Reichardt P, Chmelik P, Gaffke G, Kretzschmar A, Schneider U, Felix R, Hohenberger P (2005). Follow-up of gastrointestinal stromal tumours (GISTs) during treatment with Imatinib mesylate by abdominal MRI. Eur Radiol.

[B43] Bucher P, Egger JF, Gervaz P, Ris F, Weintraub D, Villiger P, Buhler LH, Morel P (2006). An audit of surgical management of gastrointestinal stromal tumours (GISTs). Eur J Surg Oncol.

[B44] Kosmadakis N, Visvardis EE, Tsimara M, Chatziantoniou A, Panopoulos I, Erato P, Capsambelis P (2005). The role of surgery in the management of gastrointestinal stromal tumours (GISTss) in the era of imatinib mesylate effectiveness. Surg Oncol.

[B45] Neuhaus SJ, Clark MA, Hayes AJ, Thomas JM, Judson I (2005). Surgery for gastrointestinal stromal tumour in the post-imatinib era. ANZ J Surg.

[B46] Boni L, Benevento A, Dionigi G, Rovera F, Dionigi R (2005). Surgical resection for gastrointestinal stromal tumors (GIST): experience on 25 patients. World J Surg Oncol.

[B47] Bumming P, Ahlman H, Andersson J, Meis-Kindblom JM, Nilsson B (2006). Population-based study of the diagnosis and treatment of gastrointestinal stromal tumours. Br J Surg.

